# Anaphylaxis: Five Years’ Experience in the Emergency Rooms of Five University Hospitals in Korea

**DOI:** 10.3390/medicina56120695

**Published:** 2020-12-14

**Authors:** Bo Young Chung, Ji Young Um, Jin Cheol Kim, Seok Young Kang, Min Je Jung, Hye One Kim, Chun Wook Park

**Affiliations:** Department of Dermatology, Kangnam Sacred Heart Hospital, Hallym University College of Medicine, Singil-ro, Yeoungdeungpo-gu, Seoul 07441, Korea; victoryby@naver.com (B.Y.C.); ujy0402@hanmail.net (J.Y.U.); aiekfne@naver.com (J.C.K.); tjdjrdud@naver.com (S.Y.K.); luckyminja77@naver.com (M.J.J.)

**Keywords:** anaphylaxis, epidemiology, clinical features, cause, management

## Abstract

*Background*: Anaphylaxis is an allergic disease that requires special handling due to its potential fatality. Recent epidemiological data indicate that the incidence of anaphylaxis is rising. However, actual data on the prevalence or causes of anaphylaxis in Korea are limited. *Methods*: The emergency room attendees diagnosed with anaphylaxis between 2011 and 2015 in five university hospitals were included. Medical records were reviewed retrospectively. *Results*: During the 5 years, a total of 505 subjects were diagnosed with anaphylaxis. Respiratory presentations were more common in children than in adults, while adults presented more frequently with cardiovascular symptoms. Intraoral angioedema was more often observed in the countryside than in the city. Insect stings/bites were more common in the countryside than in the city. Drugs were much more common in adults than in children. In the countryside, the frequency of anaphylaxis was higher in summer and autumn than in spring and winter. The use of corticosteroids was less common in children than in adults, while children more frequently got treatment with inhaled beta 2 agonist. *Conclusions*: The principal causes of anaphylaxis in Korean patients were food, drugs, and stings/bites. The cause, clinical features and management of anaphylaxis were significantly different depending on age and region. These real-world data on anaphylaxis could be helpful to deepen that understanding of this condition for physicians and patients.

## 1. Introduction

Anaphylactic reactions include systemic allergic reactions such as urticaria, dyspnea, hypotension, and loss of consciousness. These are explained by an IgE-mediated reaction that activates mast cells or basophils that secrete chemical media [1,2,3]. However, anaphylaxis has been used clinically in a broader sense including anaphylactic reactions that are similar to or manifested by anaphylactic reactions due to nonimmune mechanisms, as well as by immunological mechanisms [4,5]. The prevalence rate is known to be 0.05–2% [4] outside Korea, and within the country, it is reported to be 0.014% [6,7].

In domestic research, six years of data on 138 anaphylaxis patients from a university hospital in Seoul showed that the causes of anaphylaxis were drugs (34.8%), food (21.0%), unknown (13.0%), exercise (13.0%), and insect bites (11.6%) [6]. At a university hospital in Suwon, 11 years of data on 158 patients reported the causes of anaphylaxis as drugs (51.2%), insect bites (25.3%), food (10.8%), and exercise (6.3%) [7]. There are numerous causes of anaphylaxis, and there are variable clinical responses, even with the same cause [5]. This means that, in reality, the study of anaphylaxis is not easy, and there are not many reports in the literature from practice [6,7,8,9,10,11,12,13,14,15,16,17,18]. In particular, it is very important to identify the cause and risk factors, but there are a limited number of reports in Korea [6,7,9].

In this study, we examined the clinical characteristics of anaphylaxis in target patients visiting five university hospitals in Seoul, Anyang, Dongtan (Gyeonggi-do) and Chuncheon (Gangwon-do) over five years. In particular, we aimed to distinguish the differences between anaphylaxis in children and adults, as well as regional differences between urban and rural areas.

## 2. Materials and Methods

### 2.1. Study Population

Data were collected retrospectively on patients who were diagnosed with anaphylaxis between January 2011 and December 2015 in five university hospitals including Hallym University, Kangnam, Kangdong, Dongtan and Chuncheon Sacred Heart Hospital located in Seoul, Anyang, Dongtan (Gyeonggi-do) and in Chuncheon (Gangwon-do) in Korea.

### 2.2. Ethics Statement

The present study protocol was reviewed and approved by the Institutional Review Board of Kangnam Sacred Heart Hospital (approval No. HKS 2018-05-025). Informed consent was submitted by all subjects when they were enrolled.

### 2.3. Assessment Using New Criteria of Anaphylaxis

The diagnosis of anaphylaxis was defined according to the three criteria below, one of which was based on definitions reported by Simons et al. [2]. Anaphylaxis is diagnosed when any one of the following three criteria is fulfilled: (1) Sudden onset of illness (minutes to several hours), with involvement of the skin, mucosal tissue, or both. (2) When two or more of the following occur suddenly after exposure to a likely allergen or other trigger for the patients: (a) Sudden skin or mucosal symptoms and signs, (b) Sudden respiratory symptoms and signs, (c) Sudden reduction in blood pressure (BP), or symptoms of end-organ dysfunction, and (d) Sudden gastrointestinal symptoms. (3) When BP drops after exposure to a known allergen for that patient (minutes to several hours).

In accordance with the International Statistical Classification of Diseases, 10th Revision (ICD-10), patients diagnosed with anaphylaxis were targeted [11]. The ICD code used is listed in Supplement Table S1.

### 2.4. Medical Record Review

From January 2011 to December 2015, we analyzed the selected patients’ demographic characteristics and clinical modalities: (1) age of onset, (2) onset place (home, outdoor, hospital, restaurant, school, etc.), (3) duration of symptoms, (4) personal/family history of atopy, (5) history of drug eruption, (6) history of other chronic systemic diseases, such as cardiovascular disease, diabetes, respiratory diseases, and other ailments, and (7) personal/family history of anaphylaxis.

Based on the respective medical records, first, the types of symptoms were categorized. Clinical manifestations of anaphylaxis were classified into four groups: cutaneous, respiratory, cardiovascular, and gastrointestinal symptoms. The medical records of the selected patients and the laboratory results were analyzed. The treatments used, including the drug administration history and management of symptom control for each patient, were investigated.

### 2.5. Statistical Analysis

Results were expressed using mean and standard deviation (means ± standard deviation). A comparison was made between the two subgroups using a chi-square test. The statistical significance of continuous variables was confirmed by using an independent sample *t*-test (Student’s *t*-test). The Mann–Whitney U-test was used to establish difference between the two groups with no normal distribution. Statistical significance for all analyses was defined as *p* < 0.05. All statistical analyses were performed using Predictive Analysis SoftWare package version 18 (IBM SPSS, IBM North America, New York, NY, USA).

## 3. Results

### 3.1. Demographics 

**Figure 1:**
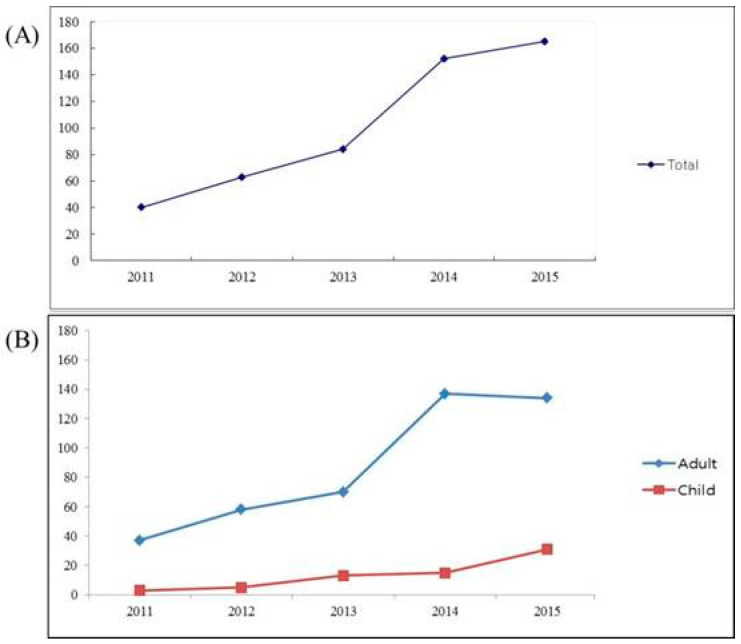
Annual number of patients with anaphylaxis observed in five university hospitals in Korea. (**A**) The total number has been increasing for five years. (**B**) There were increasing trends in both groups when classified into adult and children, but the change was more pronounced in adults.

The number of anaphylaxis episodes included during the 5-year study period was 505 at the emergency rooms of five university hospitals in Korea (Figure 1). The characteristics of study group is shown in Table 1. The number of diagnosed patients in the rural area was 23 (4.55%). The mean age of all patients was 41.0 years; 247 (48.9%) were male. There was a difference in sex between adult and children. There were more males in the children (*p* = 0.002). The presence of a personal history of allergic diseases was significantly higher in children than adults, especially asthma (*p* < 0.001).

We found that manifestations of atopic dermatitis (adult vs. children; 0.5% vs. 13.2%, *p* = 0.685), allergic rhinitis (adult vs. children; 2.3% vs. 5.8%, *p* = 0.093), and asthma (adult vs. children; 3.4% vs. 4.4%, *p* < 0.001) were higher in children with anaphylaxis than in adults with anaphylaxis. Among atopic dermatitis, allergic rhinitis, and asthma, only asthma showed statistical significance.

In rural areas, over two third of patients were over 50 years old. Among children, over 60% of patients were under 10 years old.

### 3.2. Clinical Features

The clinical manifestations of subjects are listed on Table 2. Cutaneous features were the most common clinical symptoms (85.5%). The most common symptom of 432 patients with cutaneous features was urticaria 412 (81.6%). Among the clinical manifestations, the frequency of cutaneous or oral gastrointestinal symptoms at presentation was similar for the two regions and two age groups. With regard to ages, respiratory presentations were more common in children than in adults (73.5% vs. 57.4%, *p* = 0.012), while adults presented more frequently with cardiovascular symptoms (27.7% vs. 8.8%, *p* = 0.001). The incidence of cardiovascular and respiratory presentations by age was statistically significant.

### 3.3. Causative Agents and Seasonal Distribution 

The causative agents are listed in Table 3. The causes of anaphylaxis were food (43.8%), drugs (33.3%), insect stings/bites (10.7%), exercise (1.6%) and others, in order of prevalence. In the present study, food was the most common cause. Among foods, seafood (11.9%) was the most common. The majority of stings/bites involved bees (9.7%). In terms of drugs, NSAIDs (10.3%) and antibiotics (6.5%) accounted for the majority.

In the present study, food was the most common cause in adults and children. Moreover, the causative foods were different between the two age groups. Sea food accounted for 14.7% of anaphylaxis in children and 3% in adults (*p* < 0.001). Drugs were much more common in adults than in children (37.3% vs. 7.4%, *p* < 0.001). All the patients with anaphylaxis due to sting/bites were adults (*p* = 0.002). The above results were statistically significant.

The seasonal distributions with frequency of occurrence in 505 episodes of anaphylaxis are shown in Table 1. Overall, all four seasons seemed similar, but in the case of the rural area, the frequency was higher in summer (47.8%) and autumn (39.1%). When divided into adults and children, among children, autumn (42.6%) showed the highest anaphylaxis frequency.

### 3.4. Laboratory Findings

Although there was no statistical significance, the total eosinophil count was higher in children than in adults (adult vs. children; 140.2 vs. 430.0, *p* = 0.051) (Table 1 and Table 4). The abnormal results of the various tests on the patient groups are provided in Table 4. Increased WBC counts and abnormal LFT were more frequent in children than in adults (adult vs. children; 17.5% vs. 72.4%, *p* < 0.001). Among 38 subjects who had a personal history of atopy, 17 subjects conducted total IgE level. Since most of them (15/17, 88%) showed a total IgE level above 100 IU/mL, IgE-mediated atopy was a possible cause of anaphylaxis.

### 3.5. Management of Anaphylaxis 

The majority of the patients received histamine antagonist (95.4%) or corticosteroids (95.4%) as the mainstay treatment (Table 5). Epinephrine was used in 334 patients (66.1%); whereas 97.4% were given a fluid challenge. Two (0.4%) required endotracheal intubation, and two (0.4%) underwent cardiopulmonary resuscitation. Endotracheal intubation and cardiopulmonary resuscitation were performed on one case each in the urban and the rural area. With regard to ages, the use of corticosteroids (child vs. adult; 51.5% vs. 95.2%, *p* < 0.001), intravenous fluid (child vs. adult; 88.2% vs. 98.9%, *p* < 0.001), and epinephrine (55.9% vs. 67.7%, *p* = 0.055) were less common in children than in adults, while children received treatment with inhaled beta 2 agonist more frequently (child vs. adult; 23.5% vs. 8.9%, *p* < 0.001).

## 4. Discussion

Anaphylaxis is a condition that can manifest itself in various ways, including such as dizziness, upper respiratory tract closure, low blood pressure, and cardiac arrhythmia [2,19]. In Europe and North America, the prevalence of anaphylaxis is reported to be around 8 to 60 persons per 100,000 people, and the prevalence in a lifetime is known to be around 0.05%, with increasing occurrence in younger generations [4]. The clinical characteristics of anaphylaxis in Korea are rarely available; therefore, we believed that the clinical and demographic pattern of anaphylaxis in Korea warranted investigation. We studied the clinical characteristic of patients with anaphylaxis who visited emergency rooms of five academic university hospitals in Korea. We were able to identify the causes, clinical features, and laboratory findings of anaphylaxis according to the age (adult vs. children) and region (urban vs. rural) of the patients in Korea, which had not been investigated previously. In our study, the annual number of patients with anaphylaxis observed in the emergency rooms of five university hospitals gradually increased every year, consistent with a previous report [20]. The steady increase in the incidence of anaphylaxis is similar to other countries (such as U.S.A., U.K., Spain, and Taiwan) [9,21,22] This trend could be due to the increasing prevalence of other allergic diseases and higher possibility of diagnosis for anaphylaxis. This trend will continue since Korea will become more westernized and the medical system will develop. The rates of anaphylaxis were highest in the age group 50–59 years. The median age of onset was 32.3 years old. Ye et al. reported that the mean age of adult patients with anaphylaxis in Korean was 46.0 years and that the rates of anaphylaxis were highest for those over 50 [20]. According to other studies, the incidence rates varied by age, region, time period, and within each inciting trigger [9]. For example, Lee et al. reported that anaphylaxis in Olmsted County, Minnesota (US) in 2001–2010 was most common in the 30–39 age group [9]. However, in 1990–2000, the rates of anaphylaxis in the same region were highest in the age group 0–19 years [23].

From the perspective of the leading causes of anaphylaxis, one coincidence found in previous studies and with the present study is that food is the major cause of anaphylaxis [15]. There have been some studies in other countries, such as Thailand and Taiwan, in which it was reported that the most common etiology was medication [11,13]. However, the majority of studies showed that the most common cause was food [11,13,14,17,18]. Major food allergen categories and their rates of sensitivity are variable depending on the difference in dietary habits according to race and region [5,10]. Notably, among the foods that induced anaphylaxis, different patterns were observed. In the the United States, peanuts and tree nuts were the major food allergens [12]. On the other hand, seafood is the most common cause of food-induced anaphylaxis in Thailand [18]. In our study also, seafood accounted for 11.9% of food-induced anaphylaxis.

The most common symptoms and signs in the present study involved the cutaneous system, then the respiratory, cardiovascular, and gastrointestinal systems, and finally, the oral area. This order has also been confirmed by other studies [14,16,18]. In our study, we also found that 60.9% of patients in the rural area were over fifty, so the frequencies of medical history with chronic systemic diseases were also higher than in the urban area.

The number of anaphylaxis events during all four seasons appeared to be similar in the urban area, but in the case of the rural area, the frequency was higher in summer and autumn. In the rural area, there are many outdoor activities, such as recreation and farming, that occur mainly in summer and autumn, and it seems that patterns of sightseeing in places such as Chuncheon (Gangwon-do) might also be pertinent.

There have been a few studies investigating clinical characteristics of anaphylaxis in children or adolescents. In our results, children had more respiratory symptoms (*p* = 0.012), but fewer cardiovascular symptoms (*p* < 0.001). As in this study, the cause and clinical features in children in other studies also differed from those of adult patients [13,17,24,25]. Lee et al. reported that 85.0% of Korean patients under 18 years of age who were diagnosed with anaphylaxis showed respiratory symptoms [24]. A difference in the causes of anaphylaxis by age has also been reported, even in Korea [7,24]. In our study, sea food was a more common cause in children than in adults. Drugs were a much more common cause in adults than in children. All the patients with anaphylaxis due to sting/bites were adults. Consistent with our study, drugs and insect stings are usually more common causes in adults; by comparison, food is the most common cause in children [24,26,27,28].

The guidelines of the World Allergy Organization (WAO) for approaches to anaphylaxis and treatment were modified and amended in 2012 [2]. Based on this, the therapeutic approach and actions taken for the target subjects were analyzed in this study: 95.4% of patients received antihistamines and 66.1% of patients received epinephrine, which was close to the results in previous studies [11,16]. In the cases of children, the use of inhaled beta 2 agonists was statistically significantly high because respiratory symptoms were more common.

By retrospectively investigating the clinical features, causes, and management, we better understand the major causal factors of Korean anaphylactic patients. In particular, we were able to identify the causes, clinical features and laboratory findings of anaphylaxis for “adults and children” and “urban and rural” patients in Korea, which had not been investigated previously. This study is expected to provide more accurate information about anaphylaxis to patients or physicians. In the future, large-scale prospective epidemiologic studies will be needed to show the overall condition of anaphylaxis in Korea.

Although this study was conducted at five university hospitals in the urban area and the rural area in the past five years, there are limits to how well these results show the overall present state of anaphylaxis in Korea. The rural group is much smaller than the city group to draw complete conclusions by statistical analysis. Moreover, we could not avoid underestimation. This was a retrospective study, which means that it is likely that some patients may not have been included and that some data items could be missing. Due to the way patients were recruited for this study, the true incidence of anaphylaxis could not be determined. Furthermore, when patients report that they have taken certain foods or taken any of a variety of medicines, it is not certain that the reported items are actually responsible for the anaphylactic reactions.

## 5. Conclusions

In conclusion, analysis of anaphylaxis over five years using data from five emergency hospitals in Korea showed that 505 cases of anaphylaxis in the study patients were mainly caused by food, drugs, stings/bites, and exercise. Common clinical symptoms involved the cutaneous, respiratory, circulatory, and gastrointestinal systems, and the oral area. The clinical characteristics and management of anaphylaxis were significantly different depending on age and region.

## Figures and Tables

**Table 1:** Characteristics of study group (*n* = 505).

Characteristics	Total (*n* = 505)	City (*n* = 482)	Countryside (*n* = 23)	Adult (*n* = 437)	Children (*n* = 68)	* *p*-Value
Sex, n (male/female, %)	247(48.9)/258(51.1)	231(47.9)/251 (52.1)	16 (69.6)/ 7 (30.4)	202/235 (46.2/53.8)	45/23 (66.2/33.8)	0.002
Age, y(range, median)	41.0 (0~84, 42)	40.1 (0~84, 42)	49.0 (18~84, 51)	46 (18~84, 46)	8(0~17, 7)	
Age of onset, y(range, median)	32.3 (2~66, 33)	24.8 (2~51, 25)	66 (66, 66)	36 (20~66, 33)	N.S.	
Personal history of atopy, n (%)	38(7.5)	38(7.9)	N.S.	25(5.7)	13(19.1)	<0.001
Asthma	18(3.6)	18(3.7)		2(0.5)	9(13.2)	<0.001
Allergic rhinitis	14(2.8)	14(2.9)		10(2.3)	4(5.8)	0.093
Atopic dermatitis	11(2.2)	11(2.3)		15(3.4)	3(4.4)	0.685
Accompanied symptom, n (%)						
Angioedema	227(45.0)	222(46.1)	5(21.7)	194(44.4)	33(48.5)	0.524
History of drug eruption	25(5.0)	24(5.0)	1(4.3)	24(5.5)	1(1.5)	0.155
History of chronic systemic diseases						
Cardiovascular diseases (e.g., hypertension)	56(11.1)	55(11.4)	1(4.3)	56(12.8)	N.S.	
Diabetes mellitus	17(3.4)	14(2.9)	3(13.0)	17(3.9)	N.S.	
Respiratory diseases (e.g., COPD)	0(0)	0(0)	0(0)	0	N.S.	
Others	17(3.4)	15(3.1)	2(8.7)	17(3.9)	N.S.	
History of anaphylaxis	18(3.6)	12(2.5)	6(26.1)	16(3.7)	2(2.9)	0.742
Total IgE (Average ± SD)	322.4 ± 363.6 (*n* = 122)	322.4 ± 363.6 (*n* = 122)	N.S. (*n* = 0)	441.7 ± 617.3 *(n* = 75)	299 ± 263.8 (*n* = 47)	0.425
Eosinophil count (Average ± SD)	183.0 ± 306.5	185 ± 313.4	141.1 ± 97.4	140.2 ± 129.0	430.0 ± 778.7	0.051
	(*n* = 346)	(*n* = 329)	(*n* = 17)	(*n* = 306)	(*n* = 24)	
Onset season						
Spring	132(26.1)	130(27.0)	2(8.7)	115(26.3)	17(25.0)	
Summer	109(21.6)	98(20.3)	11(47.8)	96(22.0)	13(19.1)	
Autumn	148(29.3)	139(28.8)	9(39.1)	119(27.2)	29(42.6)	
Winter	116(23.0)	115(23.9)	1(4.3)	107(24.5)	9(13.2)	

Values are presented as number (%) N.S., not sufficient (no data); Not investigated separately. COPD, Chronic Obstructive Pulmonary Disease. IgE, immunoglobulin E. Adult: 18 and over 18 years old, Children: under 18 years old. * *p*-value was calculated by chi-square test between adult and children.

**Table 2:** Characteristics of study group (*n* = 505).

Clinical Manifestation	No. of Patients (%) (*n* = 505)	City, *n* (%) (*n* = 482)	Countryside, n (%) (*n* = 23)	Adult, *n* (%) (*n* = 437)	Children, *n* (%) (*n* = 68)	* *p*-Value
Cutaneous system	432(85.5)	415(86.1)	17(73.9)	375(85.8)	57(83.8)	0.664
Urtiacaria/angioedema	412(81.6)	395(82.0)	17(73.9)	355(81.2)	57(83.8)	0.609
General pruritus	7(1.4)	7(1.5)	0	7(1.6)	0	0.293
Flushing	1(0.2)	1(0.2)	0	1(0.2)	0	0.693
Pruritus or paresthesia of the lips, axilla, hand, or feet	15(3.0)	15(3.1)	0	15(3.4)	0	0.121
Respiratory system	301(59.6)	290(60.2)	11(47.8)	251(57.4)	50(73.5)	0.012
Wheezing	29(5.7)	27(5.6)	2(8.7)	21(4.8)	8(11.8)	0.022
Dyspnea	279(55.2)	270(56.0)	9(39.1)	236(54.0)	43(63.2)	0.154
Cough	25(5.0)	24(5.0)	1(4.3)	12(2.7)	13(19.1)	<0.001
Cyanosis	6(1.2)	6(1.2)	0	0	6(8.8)	<0.001
Rhinitis	4(0.8)	4(0.8)	0	1(0.2)	3(4.4)	<0.001
Throat tightness	8(1.6)	8(1.7)	0	7(1.6)	1(1.5)	0.936
Hoarseness	7(1.4)	7(1.5)	0	6(1.4)	1(1.5)	0.949
Cardiovascular system	127(25.1)	120(24.9)	7(30.4)	121(27.7)	6(8.8)	0.001
Hypotension	63(12.5)	58(12.0)	5(21.7)	61(14.0)	2(2.9)	0.011
Presyncope	14(2.8)	14(2.9)	0	14(3.2)	0	0.134
Chest pain	28(5.5)	26(5.4)	2(8.7)	27(6.2)	1(1.5)	0.115
Syncope	54(10.7)	52(10.8)	2(8.7)	50(11.4)	4(5.9)	0.168
Tachycardia	1(0.2)	1(0.2)	0	1(0.2)	0	0.693
Oral and gastrointestinal system	125(24.8)	119(24.7)	6(26.1)	109(24.9)	16(23.5)	0.802
Emesis	0	0	0	0	0	
Nausea	46(9.1)	44(9.1)	2(8.7)	41(9.4)	5(7.4)	0.589
Abdominal cramps	36(7.1)	36(7.5)	0	31(7.1)	5(7.4)	0.938
Intraoral angioedema of buccal mucosa, tongue, palate, or oropharynx	19(3.8)	13(2.7)	6(26.1)	18(4.1)	1(1.5)	0.286
Diarrhea	11(2.2)	11(2.3)	0	11(2.5)	0	0.186
Dysphagia	1(0.2)	1(0.2)	0	1(0.2)	0	0.693
Oral mucosal pruritus	1(0.2)	1(0.2)	0	1(0.2)	0	0.693

Values are presented as number (%). * *p*-value was calculated by chi-square test between adult and children for each clinical manifestation.

**Table 3:** The cause of anaphylaxis.

Cause	No. of Patients (%) (*n* = 505)	City, *n* (%) (*n* = 482)	Countryside, *n* (%) (*n* = 23)	Adult, *n* (%) (*n* = 437)	Children, *n* (%) (*n* = 68)	* *p*-Value
Food	221(43.8)	214(44.4)	7(30.4)	191(43.7)	30(44.1)	0.949
Sea food	60(11.9)	60(12.4)	0	13(3.0)	10(14.7)	<0.001
Dry fruits	23(4.6)	23(4.8)	0	54(12.4)	6(8.8)	0.402
Beef/Chicken/pork	12(2.4)	10(2.1)	2(8.7)	1(0.2)	3(4.4)	<0.001
Egg	4(0.8)	4(0.8)	0	12(2.7)	0	0.167
Others (burger, cake, etc.)	121(24.0)	117(24.3)	6(26.1)	108(24.7)	13(19.1)	0.315
Drugs	168(33.3)	163(33.8)	5(21.7)	163(37.3)	5(7.4)	<0.001
NSAIDS	52(10.3)	50(10.4)	2(8.7)	31(7.1)	2(2.9)	0.197
Antibiotics	33(6.5)	33(6.8)	0	50(11.4)	2(2.9)	0.077
Anesthetics	1(0.2)	1(0.2)	0	0	0	
ACE inhibitors	0	0	0	1(0.2)	0	0.693
Chemotheraphy	0	0	0	0	0	
Anti-epileptic drugs	0	0	0	0	0	
Biologics	0	0	0	0	0	
Other drugs	82(16.2)	79(16.4)	3(13.0)	80(18.3)	2(2.9)	0.007
Sting/Bites	54(10.7)	41(8.5)	13(56.5)	54(12.4)	0	0.002
Bee	49(9.7)	36(7.5)	13(56.5)	49(11.2)	0	0.004
Ant	0	0	0	0	0	
Unspeficied	5(1.0)	5(1.0)	0	5(1.1)	0	0.375
Exercise-induced	8(1.6)	8(1.7)	0	7(1.6)	1(1.5)	0.936
Radiocontrast medium	7(1.4)	7(1.5)	0	6(1.4)	1(1.5)	0.949
Idiopathic	1(0.2)	1(0.2)	0	1(0.2)	0	0.693
Others/Unknown	14(2.8)	14(2.9)	0	5(1.1)	9(13.2)	<0.001

Values are presented as number (%), * *p*-value was calculated by chi-square test between adult and children.

**Table 4:** Abnormal Laboratory findings.

	Total		Adult		Children		
Parameters	Patients Evaluated, *n*	Patients with Positivity, *n* (%)	Patients Evaluated, *n*	Patients with Positivity, *n* (%)	Patients Evaluated, *n*	Patients with Positivity, *n* (%)	* *p*-Value
Increased ESR	256	28 (10.9%)	216	22 (10.2%)	40	6 (15%)	0.407
Increased CRP	286	71 (24.8%)	241	59 (24.5%)	45	12 (26.7%)	0.755
Increased ASO	18	3 (16.7%)	8	2 (25%)	10	1 (10%)	0.396
WBC > 10,000	455	133 (29.2%)	396	103 (26%)	59	30 (50.8%)	<0.001
Eosihophils > 4%	457	31 (6.8%)	397	26 (6.5%)	60	5 (8.3%)	0.608
Total IgE > 100	138	97 (70.3%)	85	60 (70.6%)	53	37 (69.8%)	0.923
ANA	0	0	0	0	0	0	
TPO Ab	2	1 (50%)	2	1 (50%)	0	0	
Anti-thyroglobulin Ab.	3	1 (33.3%)	3	1 (33.3%)	0	0	
Anti-TSH receptor Ab.	8	0	8	0	0	0	
Abnormal TFT	0	0	0	0	0	0	
Abnormal T3	69	5 (7.3%)	67	5 (7.5%)	2	0	0.688
Abnormal T4	75	1 (1.3%)	73	1 (1.4%)	2	0	0.868
Abnormal TSH	76	13 (17.1%)	74	13 (21.6%)	2	0	0.515
abnormal LFT	183	48 (26.2%)	154	27 (17.5%)	29	21 (72.4%)	<0.001

Values are presented as number (%). * *p*-value was calculated by chi-square test between adult and children. Erythrocyte sedimentation rate, ESR; c-reactive protein, CRP; anti-streptolysin O, ASO; white blood cells, WBC; anti-nuclear antibody, ANA; thyroid peroxidase, TPO; thyroid stimulating hormone, TSH; thyroid function test, TFT; liver function test, LFT.

**Table 5:** The management of anaphylaxis.

Cause	No. (%) of Patients (*n* = 505)	City, *n* (%) (*n* = 482)	Countryside, *n* (%) (*n* = 23)	Adult, *n* (%) (*n* = 437)	Children, *n* (%) (*n* = 68)	* *p*-Value
H1 antagonist or H2 antagonist	482(95.4)	462(95.9)	20(87.0)	422(99.6)	60(88.2)	0.002
Corticosteroid	451(89.3)	432(89.6)	19(82.6)	416(95.2)	35(51.5)	<0.001
Intravenous fluid	492(97.4)	469(97.3)	23(100)	432(98.9)	60(88.2)	<0.001
Epinephrine	334(66.1)	317(65.8)	17(73.9)	296(67.7)	38(55.9)	0.055
Dopamine	2(0.4)	2(0.4)	0	2(0.5)	0	0.576
Inhaled beta 2 agonist	55(10.9)	54(11.2)	1(4.3)	39(8.9)	16(23.5)	<0.001
Sodium bicarbonate	1(0.2)	0	1(4.3)	1(0.2)	0	0.693
Endotracheal intubation	2(0.4)	1(0.2)	1(4.3)	2(0.5)	0	0.576
Cardiopulmonary resuscitation (CPR)	2(0.4)	1(0.2)	1(4.3)	2(0.5)	0	0.576

Values are presented as number (%) * *p*-value was calculated by chi-square test between adult and children.

## Supplementary Materials

The following are available online at https://www.mdpi.com/1010-660X/56/12/695/s1, Table S1: ICD-10-CM diagnostic codes to obtain anaphylaxis cases included in study group.

## References

[1] Sampson H.A., Muñoz-Furlong A., Campbell R.L., Adkinson N.F., Bock S.A., Branum A., Brown S.G., Camargo C.A., Cydulka R., Galli S.J. (2006). Second symposium on the definition and management of anaphylaxis: Summary report—Second National Institute of Allergy and Infectious Disease/Food Allergy and Anaphylaxis Network symposium. J. Allergy Clin. Immunol..

[2] Simons F.E., Ardusso L.R., Bilò M.B., Dimov V., Ebisawa M., El-Gamal Y.M., Ledford D.K., Lockey R.F., Ring J., Sanchez-Borges M. (2012). 2012 Update: World Allergy Organization Guidelines for the assessment and management of anaphylaxis. Curr. Opin. Allergy Clin. Immunol..

[3] Bohlke K., Davis R.L., DeStefano F., Marcy S.M., Braun M.M., Thompson R.S. (2004). Epidemiology of anaphylaxis among children and adolescents enrolled in a health maintenance organization. J. Allergy Clin. Immunol..

[4] Simons F.E. (2010). Anaphylaxis. J. Allergy Clin. Immunol.

[5] Jang G.C., Chang Y.-S., Choi S.H., Song W.-J., Lee S.-Y., Park H.-S., Kang H.-R., Ye Y.-M., Jin H.-J., Shin M.Y. (2013). Overview of anaphylaxis in Korea: Diagnosis and management. Allergy Asthma Respir. Dis..

[6] Yang M.S., Lee S.H., Kim T.W., Kwon J.W., Lee S.M., Kim S.H., Kwon H.S., Park C.H., Park H.W., Kim S.S. (2008). Epidemiologic and clinical features of anaphylaxis in Korea. Ann. Allergy Asthma Immunol..

[7] Kim M.-J., Choi G.-S., Um S.-J., Sung J.-M., Shin Y.-S., Park H.-J., Ye Y.-M., Nahm D.-H., Lee S.-Y., Park H.-S. (2008). Anaphylaxis; 10 years‘ experience at a university hospital in Suwon. J. Asthma Allergy Clin. Immunol..

[8] Yang M.S., Kim J.Y., Kim B.K., Park H.W., Cho S.H., Min K.U., Kang H.R. (2017). True rise in anaphylaxis incidence: Epidemiologic study based on a national health insurance database. Medicine.

[9] Lee S., Hess E.P., Lohse C., Gilani W., Chamberlain A.M., Campbell R.L. (2017). Trends, characteristics, and incidence of anaphylaxis in 2001–2010: A population-based study. J. Allergy Clin. Immunol..

[10] Kim S.H., Kang H.R., Kim K.M., Kim T.B., Kim S.S., Chang Y.S., Kim C.W., Bahn J.W., Kim Y.K., Cho S.H. (2003). The sensitization rates of food allergens in a Korean population: A multi-center study. J. Asthma Allergy Clin. Immunol..

[11] Jirapongsananuruk O., Bunsawansong W., Piyaphanee N., Visitsunthorn N., Thongngarm T., Vichyanond P. (2007). Features of patients with anaphylaxis admitted to a university hospital. Ann. Allergy Asthma Immunol..

[12] Gonzalez-Estrada A., Silvers S.K., Klein A., Zell K., Wang X.F., Lang D.M. (2017). Epidemiology of anaphylaxis at a tertiary care center: A report of 730 cases. Ann. Allergy Asthma Immunol..

[13] Hsin Y.C., Hsin Y.C., Huang J.L., Yeh K.W. (2011). Clinical features of adult and pediatric anaphylaxis in Taiwan. Asian Pac. J. Allergy Immunol..

[14] Khan N.U., Shakeel N., Makda A., Mallick A.S., Ali Memon M., Hashmi S.H., Khan U.R., Razzak J.A. (2013). Anaphylaxis: Incidence, presentation, causes and outcome in patients in a tertiary-care hospital in Karachi, Pakistan. QJM.

[15] Kemp S.F., Lockey R.F., Wolf B.L., Lieberman P. (1995). Anaphylaxis. A review of 266 cases. Arch. Intern. Med..

[16] Smit D.V., Cameron P.A., Rainer T.H. (2005). Anaphylaxis presentations to an emergency department in Hong Kong: Incidence and predictors of biphasic reactions. J. Emerg Med..

[17] Orhan F., Canitez Y., Bakirtas A., Yilmaz O., Boz A.B., Can D., Kuyucu S., Harmanci K., Tahan F., Reisli I. (2011). Anaphylaxis in Turkish children: A multi-centre, retrospective, case study. Clin. Exp. Allergy.

[18] Piromrat K., Chinratanapisit S., Trathong S. (2008). Anaphylaxis in an emergency department: A 2-year study in a tertiary-care hospital. Asian Pac. J. Allergy Immunol..

[19] Lee J.K., Vadas P. (2011). Anaphylaxis: Mechanisms and management. Clin. Exp. Allergy.

[20] Ye Y.M., Kim M.K., Kang H.R., Kim T.B., Sohn S.W., Koh Y.I., Park H.K., Jang G.C., Kim C.W., Jee Y.K. (2015). Predictors of the severity and serious outcomes of anaphylaxis in korean adults: A multicenter retrospective case study. Allergy Asthma Immunol. Res..

[21] Sheikh A., Hippisley-Cox J., Newton J., Fenty J. (2008). Trends in national incidence, lifetime prevalence and adrenaline prescribing for anaphylaxis in England. J. R. Soc. Med..

[22] Tejedor-Alonso M.A., Moro-Moro M., Mosquera González M., Rodriguez-Alvarez M., Pérez Fernández E., Latasa Zamalloa P., Farias Aquino E., Gil Prieto R., Gil de Miguel A. (2015). Increased incidence of admissions for anaphylaxis in Spain 1998–2011. Allergy.

[23] Decker W.W., Campbell R.L., Manivannan V., Luke A., St Sauver J.L., Weaver A., Bellolio M.F., Bergstralh E.J., Stead L.G., Li J.T. (2008). The etiology and incidence of anaphylaxis in Rochester, Minnesota: A report from the Rochester Epidemiology Project. J. Allergy Clin. Immunol..

[24] Lee S.Y., Ahn K., Kim J., Jang G.C., Min T.K., Yang H.J., Pyun B.Y., Kwon J.W., Sohn M.H., Kim K.W. (2016). A Multicenter Retrospective Case Study of Anaphylaxis Triggers by Age in Korean Children. Allergy Asthma Immunol. Res..

[25] Seo D.-H., Ye Y.-M., Kim S.-C., Ban G.-Y., Kim J.-H., Shin Y.-S., Park H.-S., Lee S.-Y. (2016). A single hospital survey of anaphylaxis awareness among health care providers and medical students. Allergy Asthma Respir. Dis..

[26] De Silva I.L., Mehr S.S., Tey D., Tang M.L. (2008). Paediatric anaphylaxis: A 5 year retrospective review. Allergy.

[27] Huang F., Chawla K., Järvinen K.M., Nowak-Węgrzyn A. (2012). Anaphylaxis in a New York City pediatric emergency department: Triggers, treatments, and outcomes. J. Allergy Clin. Immunol..

[28] Lim D.H. (2008). Epidemiology of anaphylaxis in Korean children. Korean J. Pediatrics.

