# Supplementary material for: Anaphylaxis: Five Years’ Experience in the Emergency Rooms of Five University Hospitals in Korea
Source: Medicina (Kaunas). 2020 Dec 14;56(12):695. doi: 10.3390/medicina56120695 (PMC7764798; doi:10.3390/medicina56120695)
Supplement: Supplementary file 1 [file medicina-56-00695-s001.pdf]

Supplement table 1. ICD-10-CM diagnostic codes to obtain anaphylaxis cases included in study group

Major category and subheadings (intended)		
Anaphylactic shock, unspecified (T78.2)		
	due to adverse effect of correct drug or medicament properly administered (T88.6)	due to food (T78.0)
	due to serum (T80.5)	
Urticaria (L50.9)		
	Angioneurotic edema (T78.3)	Allergic (L50.0)
Wheezing (R06.2)		
	Status asthmaticus and Nausea and Vomiting (J46 and R11)	Wheezing and Nausea and Vomiting (R06.2 and R11)
	Status asthmaticus and Syncope and Collapse (J46 and R55)	Edema of larynx (J38.4)
Toxic effect		
	Poisoning by, adverse effect of and underdosing of insulin and oral hypoglycemic [antidiabetic] drugs (T38.3)	of venom of scorpion (T63.2)
	of venom of other arthropods (T63.4)	
